# Patient-reported continuity of care and the association with patient experience of cardiovascular prevention: an observational study in Germany

**DOI:** 10.1186/s12875-022-01788-7

**Published:** 2022-07-18

**Authors:** Christine Arnold, Patrick Hennrich, Michel Wensing

**Affiliations:** Department of General Practice and Health Services Research, Heidelberg University Hospital, Im Neuenheimer Feld 130.3, 69120 Heidelberg, Germany

**Keywords:** Family practice, Continuity of care, Patient-reported healthcare experience, Cardiovascular diseases, Cardiovascular prevention

## Abstract

**Background:**

Cardiovascular diseases are often accompanied by comorbidities, which require good coordination of care. Especially in fragmented healthcare systems, it is important to apply strategies such as case management to achieve high continuity of care. The aim of this study was to document continuity of care from the patients’ perspective in ambulatory cardiovascular care in Germany and to explore the associations with patient-reported experience of cardiovascular prevention.

**Methods:**

This cross-sectional observational study was performed in primary care practices in Germany. The study included patients with three recorded chronic diseases, including coronary heart disease. Continuity of care was measured with the Nijmegen Continuity Questionnaire, which addresses personal/relational and team/cross-boundary continuity. From aspects of medical care and health-related lifestyle counselling a patient-reported experience of cardiovascular prevention index was formed with a range of 0–7. The association between continuity of care within the family practice and patient-reported experience of cardiovascular prevention was examined, using a linear multilevel regression model that adjusted for sociodemographics, structured care programme and numbers of contacts with the family practice.

**Results:**

Four hundred thirty-five patients from 26 family practices participated. In a comparison between general practitioners (GPs) and cardiologists, higher values for relational continuity of care were given for GPs. Team/cross-boundary continuity for ‘within the family practice’ had a mean of 4.0 (standard deviation 0.7) and continuity between GPs and cardiologists a mean of 3.8 (standard deviation 0.7). Higher personal continuity of care for GPs was positively associated with patient-reported experience (b = 0.75, 95% CI 0.45–1.05, *P* < 0.001).

**Conclusions:**

Overall, there was high patient-reported continuity, which positively influenced the experience of cardiovascular prevention. Nevertheless, there is potential for improvement of personal continuity of the cardiologists and team/cross-boundary continuity between GPs and cardiologists. Structured care programs may be able to support this.

**Trial registration:**

We registered the study prospectively on 7 November 2019 at the German Clinical Trials Register (DRKS) under ID no. DRKS00019219.

**Supplementary Information:**

The online version contains supplementary material available at 10.1186/s12875-022-01788-7.

## Background

Cardiovascular diseases are the most common cause of death worldwide and are often accompanied by comorbidities, which requires good coordination of care [1, 2]. The aim of care coordination is to achieve high continuity of care, because this is associated with lower rates of hospitalisation and mortality [3, 4]. A high continuity of care also enhances patients’ feeling of security and confidence [5, 6]. According to Haggerty et al. [7], continuity of care has three dimensions: informational continuity (information sharing between different providers of information on medical history and patient preferences), relational continuity (having a trusting relationship with a healthcare provider), and management continuity (if multiple providers are involved in care, their approach is consistent with one another and congruent with patient needs). Therefore, especially in fragmented systems, such as in Germany, it is important to apply strategies such as case management, to achieve high continuity of care. It is crucial to elicit the patient’s perspective on perceived healthcare as only patients experience the full trajectory of their care episodes.

Ambulatory care in Germany, which includes primary care and specialised outpatient services, is primarily organized in single-handed practices with one to two physicians and around one to four practice assistants [8]. In 2009, general practitioner (GP)-centred care (German: “Hausarztzentrierte Versorgung”) was introduced to enforce strong general practice care and counteract fragmentation, which is specified in paragraph 73b of the German Social Code Book Five. The GP-centred care includes, for example, participation in structured care programmes for chronic conditions and referrals to specialists being exclusively coordinated by the general practitioner. Participation in these primary care programmes is optional for the patient and is offered by the statutory health insurance companies. Alternatively, patients may choose to remain in standard care, where there is a free choice of specialist without prior consultation or referral from the GP. In the structured care programmes (disease management programmes), the care for chronically ill people such as those suffering from chronic heart disease and type 2 diabetes mellitus is regulated. These programmes, e. g., include a quarterly consultation with the GP to check the state of health and progression of the respective condition, through, for example, blood pressure measurements and weight checks [9].

Following Haggerty et al. [7], Uijen et al. [10, 11] distinguish two domains in continuity of care from the patient's perspective: a) personal/relational and b) team/cross-boundary continuity. They found that patients had difficulty distinguishing between informational and management continuity, so they combined these domains as team/cross-boundary continuity.

In their study using health insurance claims data with a mixed study population from Germany, Wensing et al. [12] showed that, overall, there was a high personal continuity of care with respect to the Usual Care Provider Index (concentration of contact with a specific healthcare provider within an episode of care). In addition, participation in primary care-centred care resulted in lower hospitalisation. Vogt et al. [13] reached similar conclusions for heart failure with German claims data from 2010. With a maximum continuity of 1, they found a mean range of 0.77 to 0.89. The continuity of care indices were associated with lower hospitalisation, as was shown in logistic regression.

In addition to mortality, hospitalisation and other health outcomes, the provision of preventive medication and lifestyle counselling are important indicators of high-quality care. For example, care guidelines recommend nutrition or activity counselling [14]. For instance, the prescription of statins is mentioned as a quality indicator of good healthcare in patients with coronary heart disease [15, 16]. Studies by Ludt et al. [17] found that the documented lifestyle counselling showed that there is potential for improvement. These previous studies did not consider the role of ambulatory cardiologists, who provide cardiovascular prevention and treatment in Germany and thus need to be considered in research on care continuity and outcomes of cardiovascular care.

The aims of this study were to document patients’ perceptions of continuity of care, their experience of cardiovascular prevention in German primary and ambulatory care settings and to explore the associations between continuity of care and experience of cardiovascular care.

## Methods

### Study design and study population

This cross-sectional observational study was part of the ExKoCare project, which examined information-exchange networks in German primary care [18]. The three-year (2019–2022) project aimed to recruit a sample of 40 family practices to explore coordination and continuity of cardiovascular care in the German states of Baden-Wuerttemberg (approximately 11 million inhabitants, sampling in 10 of the 44 counties), Rhineland-Palatinate (approximately 4 million inhabitants, sampling in 13 of the 36 counties), and Saarland (approximately 1 million inhabitants, sampling in 2 of the 6 counties). The study received ethics approval from the Ethics Committee of the Medical Faculty of Heidelberg (ID: S-726/2018) and from the respective State Medical Chambers. Due to the survey being anonymous, Ethics Committee of the Medical Faculty of Heidelberg approved waiver for informed consent. Additionally, participants were informed about this in writing, as well as that returning the questionnaire is sufficient. We registered the study prospectively on 07/11/2019 at the German Clinical Trials Register under ID no. DRKS00019219. The reporting guideline ‘STROBE’ [19] for observational studies was considered in the reporting of this study (see Supplementary file 1).

The study population comprised patients with at least three recorded chronic diseases, one of them being coronary heart disease (ICD-10-GM-2022 I25.), who were registered with the participating primary care practices. We set three chronic conditions as inclusion criteria to achieve some complexity and need for care coordination.

### Data collection

Data on sociodemographics, cardiovascular care and continuity of care were collected using a written anonymised questionnaire with validated and newly developed parts. The questionnaire used a pseudonym for each practice, so that the results could be assigned accordingly. After the practice owners consented to the ExKoCare study, all practices were contacted and invited to support patient recruitment. The aim was to recruit 15 patients from each primary care practice (*n* = 600 in total). We assumed a response rate of 30%, and so we intended to invite 50 patients per practice. The research team assisted the practices via phone in identifying potential study participants to ensure that the inclusion criteria were met. A list of potential participants was compiled from the physicians' billing systems. This system lists all patients from whom services have been billed, regardless of whether they regularly visit this doctor. Then, up to 50 patients were selected by selecting every 3rd patient from a starting point specified by the researcher. If there were fewer than 50 patients on the list, all were selected. Prior to this, the physician was asked to check for the cognitive ability to complete a questionnaire and potential contra-indications. Patients were sent the questionnaire by post and an anonymous return envelope addressed to the research department was included, so that the practices did not know which patients participated.

### Measures

The questionnaire included four parts. The first part asked for personal and medical history data such as sex (male or female), age in years, health insurance (statutory health insurance, private health insurance, self-pay patient and other), chronic conditions (e.g. coronary heart disease, chronic heart failure or diabetes mellitus type 2) and participation in structured care programme (disease management programme or GP-centered care).

The second part was the validated Nijmegen Continuity Questionnaire (NCQ) [10, 11]. The NCQ was developed in The Netherlands and validated in different countries and applied in different settings (e.g., in Norway in the field of rehabilitation) [10, 20]. The 12-item NCQ includes the following subscales: personal continuity-1 (‘care provider knows me’, items 1–5), personal continuity-2 (‘care provider shows commitment’, items 6–8), and team/cross-boundary continuity (items 9–12). The two subscales for personal continuity were used to assess the continuity regarding the family doctor and cardiologist and the team continuity for family practice (continuity between physicians and practice assistants) and the cross-boundary continuity between family doctor and cardiologist. Thus, the questionnaire contained six continuity of care scores and a total of 24 items.

Each question could be answered on a 5-point Likert scale: 1 (*strongly disagree*), 2 (*disagree*), 3 (*neutral*), 4 (*agree*) to 5 (*strongly agree*), or with *I do not know*. The English version of all 12 items in the NCQ was translated carefully and independently by CA and PH using a forward translation into German and a backwards translation into English. After the independent translations, consensus discussions were held and a common German version was produced. A test with interviews in 6 patients did not identify significant lack of clarity.

Confirmatory factor analysis was used to examine the postulated clustering of items for the general practice and cardiologist domains. The analysis for general practitioners had a comparative fit index of 0.95, a root mean square error of approximation of 0.10, and a standardised root mean square residual of 0.04. The analysis for cardiologists showed a comparative fit index of 0.92, a root mean square error of approximation of 0.13, and a standardised root mean square residual of 0.05. The comparative fit index for both healthcare providers confirmed a good fit.

The last two sections of the self-administered questionnaire referred to cardiovascular care. Patient-reported numbers of cardiovascular contacts in the last three months with different healthcare professionals (e. g. general practitioners, cardiologists and nurses). The patients were also asked whether they had been treated regularly by one or more GP.

Questions on patient-reported experience of cardiovascular prevention during the past 12 months were derived from the two National Health Care Guidelines for chronic coronary heart disease and heart failure and from quality indicators of ambulatory care in Germany [14, 15, 21]. Content validity was assured by careful deduction of indicators from key aspects of prevailing clinical guidelines on cardiovascular prevention. In addition, an analysis of missing values was performed to provide a measure of the comprehensibility of the questionnaire. From aspects of medical care and health-related lifestyle counselling in the past 12 months (advice on physical exercise, advice on weight, advice on eating habits, discussion of therapeutic goals and medication plan, handing out of informational material about heart disease and the prescription of statins) a patient-reported experience of cardiovascular prevention index was formed with a range of 0–7. All of the seven items were weighted equally. The maximum value of 7 indicates that the patient has received all lifestyle counselling and medical care in the past 12 months. More items answered with ‘yes’ were interpreted as better experience of cardiovascular prevention.

### Data analysis

The paper-based questionnaires were scanned and imported into a computer file. All analyses were performed in R (version 4.0.5) using R Studio (version 1.2.5033).

First, patient characteristics, contacts with various healthcare providers and items of patient-reported experience of cardiovascular prevention were analysed. The descriptive analysis included frequencies and percentages for categorical variables and mean with standard deviation (SD) or median with interquartile range (IQR) for continuous variables according to distribution. Categorical variables were tested for independence with regard to inclusion in the final regression analysis using Chi^2^-tests. For the metric variables, a t-test was performed accordingly.

To calculate the scores of ‘continuity of care’, cases were excluded if more than one item within each scale was missing and afterwards the mean with SD per subscale was computed. We did not perform imputation because of the possibility that a missing value meant no contact with a physician, which meant no assessment was possible. The difference between the personal continuity of care of the GP and the cardiologist was calculated using the t-test for independent samples. The independent samples t-test was also used to compare the subscale team/cross-boundary continuity within the GP practice (between the GP and the practice assistant) and outside the GP practice (between GP and cardiologist).

Relationships between patient-reported experience of cardiovascular prevention and the reported continuity of care were explored using the Pearson product-moment correlation. A correlation coefficient between 0.3 and 0.5 was considered as moderate and > 0.5 as a strong correlation [22].

Finally, due to the hierarchical structure of the data, we performed a linear multilevel analysis with the dependent variable ‘patient-reported experience of cardiovascular prevention score’ and the independent variable continuity of care. The final model was adjusted at level 1 for age, sex, numbers of chronic conditions, participation in disease management-programme and for numbers of contacts to GP. The second level was made up of the GP practices. In the first step of the final multilevel analysis, we calculated the null model without predictors and derived the intra-class correlation from it. In the second model, the predictors were inserted as fixed effects, thus creating a random intercept model. Restricted maximum likelihood was used to estimate the parameters.

The final model, which is presented in the results section, was selected on the basis of multicollinearity, model fit, content-related aspects, and the number of cases. We assumed multicollinearity if the correlation coefficient was greater than 0.6. The goodness of fit of the regression models was measured using the Akaike Information Criterion (AIC). To decide on the final model based on these characteristics, various regression analyses were performed. The significance level was set at α = 0.05 for all analyses.

## Results

Data were collected from November 2020 to December 2021. As planned, 42 primary care practices were recruited. However, 16 practices did not agree to patient recruitment, which seemed related to the workload that was induced by the COVID-19 pandemic. Patient identification was difficult due to the partly incomplete coding of the diseases. It was not possible to identify 50 patients in all practices. The 26 participating practices sent 811 questionnaires to patients (*n* = 31 on average). Due to missing values Sat the predictors, only 247 cases could be included in the final regression model.

A total of 435 patient questionnaires from 26 family practices were returned, giving a response rate of 53.6% and on average 16.7 responding patients (1 to 33 patients) per practice. Table 1 shows key patient characteristics. Of the 435 participants, 316 (73.0%) were male and the mean age was 74.7 (SD 9.0) years. Furthermore, only 56.1% of the patients reported chronic coronary heart disease, although this disease was recorded for all patients by physicians. Patients reported a mean of 2.0 (SD 1.0, range 0–7) heart diseases and 1.7 (SD 1.5, range 0–9) co-morbidities. Ninety-four participants did not indicate comorbidities.

**Table 1:** Patient characteristics

**Characteristics**	**Total** n (%) *N* = 435	**Sample final model** n (%) *n* = 247	**Excluded patients** n (%) *n* = 188	***P*-value**
	*n* = *406*	*n* = *247*	*n* = *159*	0.04 ^a^
**Age in years**, mean (SD)	74.7 (9.0)	73.9 (8.9)	76.0 (8.9)	
**Sex**	*n* = *433*	*n* = *247*	*n* = *186*	0.49
Female	117 (27.0)	63 (25.5)	54 (29.0)	
Male	316 (73.0)	184 (74.5)	132 (71.0)	
**Health insurance**	*n* = *426*	*n* = *240*	*n* = *186*	0.05
Statutory health insurance	380 (89.2)	220 (91.7)	160 (86.0)	
Private health insurance	39 (9.2)	16 (6.7)	23 (12.4)	
Self-pay patient	4 (0.9)	1 (0.4)	3 (1.6)	
Other	3 (0.7)	3 (1.2)	0 (0.0)	
**Cardiovascular diseases**	*n* = *435*	*n* = *247*	*n* = *188*	
Hypertension	281 (64.6)	157 (63.6)	124 (66.0)	0.68
Cardiac arrhythmia/atrial fibrillation	130 (29.9)	78 (31.6)	52 (27.7)	0.44
Coronary heart disease	244 (56.1)	147 (59.5)	97 (51.6)	0.12
Chronic heart failure	67 (15.4)	48 (19.4)	19 (10.1)	0.01 ^a^
Stroke	40 (9.2)	22 (8.9)	18 (9.6)	0.94
Peripheral vascular disease	41 (9.4)	21 (8.5)	20 (10.6)	0.56
Aortic aneurysm	17 (3.9)	7 (2.8)	10 (5.3)	0.28
Other	36 (8.3)	22 (8.9)	14 (7.4)	0.71
**Chronic diseases**	*n* = *435*	*n* = *247*	*n* = *188*	
Joint diseases	155 (35.7)	87 (35.4)	68 (36.2)	0.94
Chronic back pain	114 (26.3)	62 (25.2)	52 (27.8)	0.62
Diabetes mellitus type 2	151 (35.0)	97 (39.9)	54 (28.7)	0.02 ^a^
Chronic kidney diseases	46 (10.6)	20 (8.2)	26 (13.8)	0.08
Chronic lung diseases	54 (12.4)	32 (13.0)	22 (11.7)	0.79
Chronis thyroid diseases	54 (12.4)	29 (11.8)	25 (13.3)	0.75
Chronic gastrointestinal diseases	21 (4.8)	11 (4.5)	10 (5.3)	0.86
Allergies or skin diseases	44 (10.1)	26 (10.6)	18 (9.6)	0.86
Depression or anxiety disorders	34 (7.9)	20 (8.1)	14 (7.5)	0.95
Cancer	42 (9.7)	25 (10.2)	17 (9.0)	0.82
Other chronic diseases	24 (5.5)	15 (6.1)	9 (4.8)	0.70
**Physical activities**	*n* = *415*	*n* = *238*	*n* = *177*	0.63
Daily	90 (21.7)	49 (20.6)	41 (23.2)	
3–6 times/week	122 (29.4)	70 (29.4)	52 (29.4)	
1–2 times/week	128 (30.8)	75 (31.5)	53 (29.9)	
Less regularly, about once a month	39 (9.4)	26 (10.9)	13 (7.3)	
Never	36 (8.7)	18 (7.6)	18 (10.2)	
**Participation in HZV**	204 (57.1)	131 (60.4)	73 (52.1)	0.15
	*n* = *357*	*n* = *217*	*n* = *140*	
**Participation in DMP**	204 (53.1)	137 (55.7)	67 (47.1)	0.22
	*n* = *384*	*n* = *246*	*n* = *138*	
**Participation in HZV and DMP**	138 (31.7)	97 (39.3)	41 (21.8)	< 0.001 ^a^
	*n* = *435*	*n* = *247*	*n* = *188*	

*SD* Standard deviation, *HZV* German: Hausarztzentrierte Versorgung, Family centered care

*DMP* Disease management programme for coronary heart diseases

^**a**^ Statistically significant at α = 0.05, comparison final sample and sample of excluded patients

In terms of care, 384 (90.8%) patients reported one usual GP, while 39 (9.2%) being seen by two GPs on a regular basis. Table 2 presents information about the patients’ contact with different healthcare providers. Patients reported having the most contact with pharmacists (median 2 IQR 1–3), followed by GPs and practice assistants in family practices (median 1 IQR 0–2).

**Table 2:** Contacts with healthcare providers in the last 3 months because of cardiovascular disease

	**Total** N	**No contact** n (%)	**Contact** n (%)	**Number of contacts** Median (IQR)
Pharmacy	403	71 (17.6)	332 (82.4)	2 (1–3)
General practitioner	408	111 (27.2)	297 (72.8)	1 (0–2)
Practice assistant family practice	382	118 (30.9)	264 (69.1)	1 (0–2)
Cardiologist	389	220 (56.6)	169 (43.4)	0 (0–1)
Practice assistant cardiology practice	362	241 (66.6)	121 (33.4)	0 (0–1)
Other specialists	385	282 (73.2)	103 (26.8)	0 (0–0)
Podiatry practices	391	282 (72.1)	109 (27.9)	0 (0–1)
Classes for cardiology related exercises	383	355 (92.7)	28 (7.3)	0 (0–0)
Ambulatory nursing services	391	370 (94.6)	21 (5.4)	0 (0–0)

*IQR* Interquartile range

Table 3 summarises patient-reported continuity of care. In a comparison between GPs and cardiologists, higher values for relational continuity of care were given for GPs. A higher degree of team/cross-boundary continuity was reported within the GP practice compared to outside the GP practice. Across all items, there were 18.5% missing values or the patients could not make a response. 190 people completed the NCQ in full. Data regarding the continuity of care of the subsample of the final regression analysis can be found in the appendix (see Supplementary file 2). There was no statistically significant difference to the overall sample.

**Table 3:** Subscales scores (range 1–5) of Nijmegen Continuity Questionnaire reported by patients with cardiovascular diseases

	**General practitioner** mean (SD)	**Cardiologist** mean (SD)	***P*-value**
**Personal continuity**			
	*n* = *411*	*n* = *322*	
Care provider knows me	4.1 (0.7)	3.5 (0.8)	< 0.001
	*n* = *415*	*n* = *322*	
Care provider shows commitment	3.9 (0.8)	3.2 (1.0)	< 0.001
	**Within family practice** mean (SD) *n* = *345*	**Between GP & cardiologist** mean (SD) *n* = *305*	
**Team/cross-boundary continuity**	4.0 (0.7)	3.8 (0.8)	< 0.001

*GP* General practitioner

Table 4 provides an overview of patient-reported healthcare experience of cardiovascular prevention during the past 12 months. The body weight check was the care item most often recorded by patients (247 (58.0%)). In contrast only 90 (21.6%) stated that they had discussed their therapy goals and 24 (5.6%) that they had discussed their medication plan with their GP. Comparison between the final sample of the regression analysis and the excluded cases showed that the variables ‘advice on exercises’ and ‘talk about eating habits’ differed statistically significant between both groups. Patients from the final analysis-sample had answered ‘yes’ to the items more often.

**Table 4:** Patient-reported healthcare experience of cardiovascular prevention during the past 12 months

**Received counselling on health-related lifestyle**	**Total** n (%**)** *N* = 435	**Sample final model** n (%) *n* = 247	**Excluded patients** n (%) *n* = 188	***P*-value**
**Advice on exercises**	*n* = *418*	*n* = *241*	*n* = *177*	< 0.01 ^a^
No	242 (57.9)	125 (51.9)	117 (66.1)	
Yes	176 (42.1)	116 (48.1)	60 (33.9)	
**Advice on smoking**	*n* = *419*	*n* = *239*	*n* = *180*	0.54
No	21 (5.0)	12 (5.0)	9 (5.0)	
Yes	30 (7.2)	20 (8.4)	10 (5.6)	
I don’t smoke	368 (87.8)	207 (86.6)	161 (89.4)	
**Advice on alcohol drinking**	*n* = *420*	*n* = *241*	*n* = *179*	0.81
No	157 (37.4)	93 (38.6)	64 (35.8)	
Yes	22 (5.2)	13 (5.4)	9 (5.0)	
I don’t drink alcohol	241 (57.4)	135 (56.0)	106 (59.2)	
**Check body weight and advice accordingly**	*n* = *426*	*n* = *244*	*n* = *182*	0.07
No	179 (42.0)	93 (38.1)	86 (47.3)	
Yes	247 (58.0)	151 (61.9)	96 (53.7)	
**Talk about eating habits**	*n* = *427*	*n* = *246*	*n* = *181*	0.04 ^a^
No	294 (68.9)	159 (64.6)	135 (74.6)	
Yes	133 (31.1)	87 (35.4)	46 (25.4)	
**Receive information material**	*n* = *416*	*n* = *242*	*n* = *174*	0.70
No	240 (57.7)	142 (58.7)	98 (56.3	
Yes	176 (42.3)	100 (41.3)	76 (43.7)	
**Therapeutic goals**	*n* = *416*	*n* = *240*	*n* = *176*	0.70
No	326 (78.4)	186 (77.5)	140 (79.5)	
Yes	90 (21.6)	54 (22.5)	36 (20.5)	
**Statin**	*n* = *421*	*n* = *243*	*n* = *178*	0.10
No	76 (18.1)	37 (15.2)	39 (21.9)	
Yes	345 (81.9)	206 (84.8)	139 (78.1)	
**Medication plan**	*n* = *430*	*n* = *245*	*n* = 185	0.34
Yes, I have a medication plan and talked about it with my GP	24 (5.6)	12 (4.9)	12 (6.5)	
Yes, I have a medication plan and did not talk about it with my GP	338 (78.6)	198 (80.8)	140 (75.7)	
No, I don’t have a medication plan	68 (15.8)	35 (14.3)	33 (17.8)	

GP General practitioner

^**a**^ Statistically significant at α = 0.05, comparison final sample and sample of excluded patients

Patient-reported experience of cardiovascular prevention score (range 0–7) had a mean of 3.5 (SD 1.7) in the study population. Pearson correlation between healthcare score and continuity of care within the GP practice was *r* = 0.29, *P* < 0.001 and between healthcare score and personal continuity (‘GP shows commitment’) *r* = 0.37, *P* < 0.001. Table 5 presents the results of final multilevel regression analyses for patient-reported experience of cardiovascular prevention score: adjusted for all other variables sex, age and numbers of contacts with GP were not statistical significantly associated with the experience of cardiovascular prevention score. Due to multicollinearity, we were able to include only 3 of the 6 continuity of care scales in the final regression model (see Supplementary file 3). In the personal continuity domain, we chose the second subscale of the GPs, because GPs are primarily involved in the preventive care interventions and the model with the second subscale (“care provider shows commitment”) had a better model fit compared to the model with the first subscale (“care provider knows me”). The two team/cross-boundary continuity were selected, because they represent the coordination of different healthcare provides. Higher personal continuity of care for the GP was positively associated with the healthcare experience of cardiovascular prevention score (b = 0.75, 95% CI 0.45–1.05, *P* < 0.001). The goodness of fit of the final model with an AIC of 942 was better than the null model with 1,700. The interclass correlation was 0.08.

**Table 5:** Final multilevel model with patient-reported healthcare experience of cardiovascular prevention as dependent variable (*n* = 247 patients, 26 GP practices)

	**B-coefficient**	**Standard Error**	**95% CI**	***P*-value**
**Personal continuity GP**				
*Care provider shows commitment*	**0.75**	0.15	0.45–1.05	** < 0.001**^**a**^
**Team/cross-boundary continuity**				
*within GP practice*	0.17	0.18	–0.18–0.52	0.35
*between GP and cardiologist*	0.09	0.15	–0.21–0.38	0.57
Sex (ref. female)	0.10	0.23	–0.35–0.56	0.66
Age in years	0.002	0.01	–0.02–0.02	0.85
Number of chronic diseases	**0.17**	0.07	0.03–0.32	**0.02**^**a**^
Participation in DMP (ref. “no”)	**0.46**	0.21	0.05–0.86	**0.03**^**a**^
Numbers of contacts with GP	0.02	0.01	–0.01–0.03	0.24
Intercept	–1.01	1.15	–3.28–1.25	0.38

*CI* Confidence interval, *DMP* Disease management programme, *GP* General practitioner, *ref* Reference group

^**a**^ Statistically significant at α = 0.05

## Discussion

This study found that patients perceived high personal and team/cross-boundary continuity of general practice care, which was somewhat higher than the continuity of ambulatory cardiology care. Higher personal continuity was associated with higher patient-reported experience of cardiovascular prevention, independent of patients’ number of contacts in primary care. The study adds to the accumulating evidence on the benefits of high continuity of primary care [23–25].

Our findings are in line with a previous study with administrative data, which reported a high continuity of care in Germany in comparison with other countries e. g. United Kingdom [12]. This underlines that GPs have a core function in the care of patients with cardiovascular diseases and, combined with the relatively high number of contacts in German primary care, allows patients and physicians to know each other well. Since 2009, GP-centered care exists in Germany [8, 9], which assigns a pilot function to GPs. For example, as a result of GP-centered care, referrals to medical specialist are managed by GPs, so they always know the patients’ conditions and uncontrolled visits to specialists are avoided. In the present study, a little more than half of the participants stated that they took part in this programme.

The personal continuity of care was rated higher for GPs than for cardiologists, which was expected due to the primary care physician's role as the primary point of contact for patients. These results are consistent with studies from other countries and specialties. In the study of Cohen Castel et al. 2018 [26], patients reported a mean of 3.8 (SD 1.0) for personal continuity (‘care provider knows me’) for GPs and compared to 3.5 (1.0) for oncologists. In Uijen et al. 2012 [10], patients reported a mean of 3.7 for GPs and for various specialists 3.6. The population included patients with various chronic diseases.

The questions regarding cardiologists were answered by significantly fewer people compared to the questions concerning the GP. In addition, 56.6% reported no contact with a cardiologist during the last three months. Presumably, for these patients (recruited in general practices) contacts with cardiologists were too infrequent to answer the questions. Here, the survey of the annual contact would have been interesting, as this is also recommended in the guidelines [14, 21, 27]. Due to the phrasing of our questionnaire, we could not differentiate between whether the guideline recommendations for the involvement of cardiologists were not adhered to, or whether there was simply no contact in the past three months, but still a contact in the past year as recommended by the guidelines.

As already published in earlier studies by Ludt et al. [17, 28], the lifestyle counselling and patient-reported experience of care showed that there is potential for improvement. In our study, about half of the patients received lifestyle counselling in the past 12 months. Especially in the area of the patient's participation in their disease and therapy, there is still great potential. Therapy goals and medication schedules were only discussed between GPs and patients in less than a quarter of cases. The lack of information can also be seen in the results on heart diseases: only slightly more than half of the patients stated a coronary heart disease, while all of them were diagnosed with it by their physician according to the ICD codes (International Statistical Classification of Diseases and Related Health Problems) that were used for recruitment. In contrast, good conversion to statin therapy was seen in 81% of patients, which is close to the 85% considered as a quality indicator and to other study findings [15, 29]. Although many patients take statins, they do not seem to be informed that they have a coronary heart disease.

Our study showed a positive influence of comorbidities on experience of care. It is possible that sicker patients in particular are receiving more preventive care due to their higher physician contacts. In contrast, there is a greater need for coordination in the presence of comorbidities [30]. The study demonstrated the positive association between coordinated care as measured by personal continuity for GPs as well as participation in a structured care programme and patient-reported experience of care. This is consistent with other studies [31, 32]. Explanatory mechanisms may be that by establishing a doctor-patient relationship, trust is strengthened and the doctor's sense of responsibility increases, and appropriate preventive measures are taken to delay the progression of coronary heart disease [33]. Several trends in healthcare may reduce personal continuity, for instance ongoing medical specialization and increasing emphasis on work-life balance of physicians. Continuity of care may also be achieved in teams and networks of healthcare providers, but it needs careful design to make sure that its effects on healthcare performance and health outcomes are not lost.

### Strengths and limitations

A strength of the study was the high response rate of 53.6%. The number of missing items of the NCQ was moderate with 18.5%. However, the effective sample size in the regression analysis was reduced by missing values, especially in the questions on cardiologists. The comparison between the final sample of the regression analysis and the excluded cases showed that there is a potential selection bias with regard to patient-reported cardiovascular prevention, which must be considered in the interpretation of the results. It cannot be excluded that the positive correlation between continuity of care and cardiovascular prevention resulted from this bias. Another limitation is the extended recruitment time due to the COVID-19 pandemic. Overall, this makes the interpretation of the results difficult, as it is unclear whether there were fewer contacts with the providers due to the pandemic and whether this was only a snapshot or also existed before and after the pandemic. Unfortunately, we failed to ask if the treatment was with different GPs within one practice or different ones. This would have been additional information to assess continuity of care, as there is presumably a higher exchange of information within one practice. During recruitment, some physicians noticed that their ICD codes were not consistently documented – this possibly led to a selection bias. In addition, we cannot make any explicit statements about the number of patients with coronary heart diseases in the participating practices. Around 2,000 patients with coronary heart diseases could be identified from 24 practices of which around 800 were invited to the study. Two practices could not provide any information on this. Furthermore, there is the opportunity of social desirability bias, as it can be assumed that patients want to rate their physicians positively. In addition, only 7.2% *(n* = *30)* reported positive smoking status, which could also indicate social desirability bias. It would also have been interesting to perform a subgroup analysis for smokers to determine the experience of care they receive, since smoking status can be seen as a predictor of coronary heart disease. However, this was not possible due to the number of cases. For a better interpretation of the results, it would have been helpful to monitor the duration and severity of the heart disease. Not all parts of the questionnaire were validated, however, aspects were derived from guidelines and already used in previous larger projects, for example in the EPA-project [34].

## Conclusions

In this study, a high continuity of care could be shown especially regarding GPs, which was associated with higher patient-reported experience of cardiovascular prevention. Structured care programs could possibly help to increase continuity in the area of team/cross-boundary continuity and thus improve the experience of care.

## Supplementary Information

**Additional file 1.** Reporting guideline.

**Additional file 2.** Continuity of Care.

**Additional file 3.** Correlation matrix Continuity of Care (*n* = 247).

## Data Availability

The datasets generated and analysed during the current study are not publicly available but are available from the corresponding author on reasonable request. This is due to ongoing research and our promise to the participants to only share aggregated data in terms of manuscripts and conference presentations.

## Abbreviations

AIC: Akaike information criterion
CI: Confidence interval
COVID-19: Coronavirus disease 2019
DMP: Disease management programme
GP: General practitioner
ICD: International Statistical Classification of Diseases and Related Health Problems
IQR: Interquartile range
NCQ: Nijmegen Continuity Questionnaire
Ref: Reference group
SD: Standard deviation
