# Supplementary material for: Patient-reported continuity of care and the association with patient experience of cardiovascular prevention: an observational study in Germany
Source: BMC Prim Care. 2022 Jul 18;23:176. doi: 10.1186/s12875-022-01788-7 (PMC9289649; doi:10.1186/s12875-022-01788-7)
Supplement: Supplementary file 2 — Additional file 2. Continuity of Care. [file 12875_2022_1788_MOESM2_ESM.docx]

**Supplementary file 2. Continuity of Care**

|  | **General practitioner**  mean (SD) | **Cardiologist**  mean (SD) | **P-value** |
| --- | --- | --- | --- |
| **Personal continuity**  Care provider knows me | 4.1 (0.6) | 3.6 (0.7) | < 0.001 |
| Care provider shows commitment | 3.9 (0.8) | 3.2 (0.9) | < 0.001 |
|  | **Within family practice**  mean (SD) | **Between GP & cardiologist**  mean (SD) |  |
| **Team/cross-boundary continuity** | 4.0 (0.7) | 3.8 (0.7) | < 0.001 |

*Subscales scores (range 1–5) of Nijmegen Continuity Questionnaire reported by patients with cardiovascular diseases for subsample of the regression analysis (n= 247)*
