# Supplementary material for: Patient-reported continuity of care and the association with patient experience of cardiovascular prevention: an observational study in Germany
Source: BMC Prim Care. 2022 Jul 18;23:176. doi: 10.1186/s12875-022-01788-7 (PMC9289649; doi:10.1186/s12875-022-01788-7)
Supplement: Supplementary file 3 — Additional file 3. Correlation matrix Continuity of Care (n = 247). [file 12875_2022_1788_MOESM3_ESM.docx]

**Supplementary file 3. Correlation matrix Continuity of Care (n = 247)**

| **Variable** | **1** | **2** | **3** | **4** | **5** | **6** |
| --- | --- | --- | --- | --- | --- | --- |
| **1. Healthcare score** |  |  |  |  |  |  |
| **2. NCQ_P1_GP** | 0.25^**^ |  |  |  |  |  |
| **3. NCQ_P2_GP** | 0.44^**^ | **0.69^**^** |  |  |  |  |
| **4. NCQ_within_GP** | 0.29^**^ | 0.50^**^ | 0.56^**^ |  |  |  |
| **5. NCQ_P1_Cardio** | 0.23^**^ | 0.39^**^ | 0.37^**^ | 0.34^**^ |  |  |
| **6. NCQ_P2_Cardio** | 0.14^*^ | 0.29^**^ | 0.40^**^ | 0.38^**^ | **0.78^**^** |  |
| **7. NCQ_GP_Cardio** | 0.24^**^ | 0.36^**^ | 0.40^**^ | 0.46^**^ | **0.68^**^** | **0.67^**^** |

^*^ *p* < 0.05, ^**^ *p* < 0.01

*NCQ = Nijmegen Continuity Questionnaire, GP = general practitioner, Cardio = cardiologist*

*Healthcare score = patient-reported experience of cardiovascular prevention (range 0–7)*

*NCQ_P1_GP = Personal continuity: care provider knows me (general practitioner)*

*NCQ_P2_GP = Personal continuity: care provider shows commitment (general practitioner)*

*NCQ_within_GP = Team/cross-boundary continuity: within family practice*

*NCQ_P1_Cardio = Personal continuity: care provider knows me (cardiologist)*

*NCQ_P2_Cardio = Personal continuity: care provider shows commitment (cardiologist)*

*NCQ_GP_Cardio = Team/cross-boundary continuity: between general practitioner & cardiologist*
